# Supplementary material for: Are carbon emissions trading and green financial instruments synergistic? -Comprehensive quantitative research based on content analysis
Source: PLoS One. 2024 Mar 7;19(3):e0298601. doi: 10.1371/journal.pone.0298601 (PMC10919601; doi:10.1371/journal.pone.0298601)
Supplement: S1 Data — (ZIP) [file pone.0298601.s001.zip › Data/地级市Z_F工作报告.zip!/╣π╢1⁄2/╘╞╕í╩╨/└┤╫╘╣1⁄2╓┌║┼í╛┬φ┐╦╩2╛▌═°í┐_2004.docx]

各位代表：

我代表市人民政府向大会作政府工作报告，请予审议，并请政协各位委员和其他列席人员提出意见。

过去五年工作回顾

1999年换届以来，市政府在省委、省政府和市委的正确领导下，在市人大常委会和市政协的监督支持下，高举邓小平理论伟大旗帜，全面贯彻“三个代表”重要思想，坚持以“一城崛起，三线带动，五星争辉”的战略目标和走珠三角经济区延伸发展路子的战略措施统揽全局，以发展为主题，以体制创新为动力，以云浮实际为依据，以解决经济和社会发展过程中的重点难点问题为突破口，创新务实，艰苦奋斗，促进了全市经济和社会健康、协调发展，圆满完成了二届人大一次会议提出的各项目标任务。

——经济进入加速发展阶段。2003年，全市完成地方生产总值214.73亿元，比1998年增长46.9%，5年平均增长8%。工业总产值306.4 亿元，比1998年增长66.9 %，5年平均增长10.8 %;农业总产值96.8亿元，比1998年增长33.1%，5年平均增长5.9%;第三产业增加值66.3亿元，比1998年增长49.4%，5年平均增长8.4%;地方财政一般预算收入5.9亿元，比1998年增长85.1%，5年平均增长13.1%，其中2002、2003年增幅连续两年在全省地级市中排第1位；城乡居民储蓄存款余额131亿元，比1998年增长81.9%；国税、地税收入共11.7亿元，比1998年增长96%，5年平均增长19.2%。五年来，我市经济发展逐年加快，特别是去年地方生产总值增幅达到13.1%，排在全省山区市前列。

——走珠三角经济区延伸发展路子取得明显成效。五年来，我市积极实施走珠三角经济区延伸发展路子，大力吸引珠三角及其他经济发达地区的资金、项目和人才，使全市经济和社会发展呈现前所未有的好势头。去年，全市在建或已签约项目投资总额380多亿元，其中工业项目投资270多亿元。这些投资主要来自珠三角地区和海外，这对加速我市经济发展步伐，将起到十分重要的作用。

——各项改革取得阶段性成果。我市以建设廉洁高效政府为重点，全面推进各项改革，并取得了阶段性成果。1999年，云安、云城精简压缩了党政机关人员共1662人。在2001年开展的机构改革中，全市又精简了行政机构和人员编制20%，并实行中层干部竞争上岗，规范了公务员管理。2003年的农村税费改革，取消一批涉农收费项目，农民人均减负率达85.4%，撤并镇的各项配套措施得到落实。通过行政审批制度改革，对市直部门的913项审批项目进行了三次清理，减少了297项。率先在公用事业服务项目中引入招标竞争机制，降低了管理成本，提高了服务质量。党政机关与所办经济实体已按规定脱钩。按规定应进行招投标的建设项目、土地和矿业权出让招投标率均达100%。市、县均设立了行政服务中心，实行“一站式”服务，提高了办事效率。全面推进国有企业改革，改革面达93%，国有资产得到有效盘活。社会保障事业取得较大进展，参加养老保险人数达16.5万人，比1998年增加了5.7万人。积极推进农村医疗改革，新型农村合作医疗覆盖率居全省第1位。住房制度改革依照政策得到较好实施。

——农业结构进一步优化。我市大力推进农业产业化进程，致力发展优质、高效、生态和安全农业。“公司+农户”的温氏模式进一步推广，农业龙头企业从1998年32家增加至67家（其中国家级1家，省级13家），辐射带动的农户由5.5万户增至17.2万户，占全市农户总数的32%。城郊型农业加速发展，其中花卉苗木基地从无到有，已发展到2.1万亩，成为新的经济增长点。坚持科技兴农，建立了省级农业现代化示范区和华南农业大学农业产业化云浮实验区，获国家农业名牌产品奖4个，国家绿色食品认证产品5个，省级无公害生产基地认证11个。全市组建农民专业协会12个。罗定市、新兴县和郁南县分别被认定为中国肉桂之乡、中国果品加工之乡和中国无核黄皮之乡。劳务经济进一步发展，全市常年外出务工经商农民达31.3万人。

——工业立市步伐加快。我市在积极推广先进适用技术提升传统产业，增强竞争力，提高企业经济效益的基础上，大力发展新兴工业，推动全市工业上了一个新台阶。目前，我市的传统产业已初步形成企业集群，去年，石材工业产值40多亿元、不锈钢制品产值30多亿元、纺织服装产值20多亿元、水泥生产能力达到685万吨，被国家评为中国石材基地中心、中国石材流通示范基地，新兴县被评为中国不锈钢餐厨具之乡。通过承接珠三角产业转移，新发展了电子、陶瓷、碳酸钙、钛白粉等一批新企业，既促进工业结构优化，又提高了我市工业的技术含量，加快了全市工业化步伐。

——第三产业逐步兴起。经过几年的努力，第三产业占地方生产总值的比重明显上升，住房、电信、教育、保健、汽车已成为消费新热点，物流业逐步发展。旅游资源开发步伐加快，新上马及续建旅游建设项目17个，已投入资金2.8亿元。

——民营经济进一步壮大。我市把民营经济的发展作为富民强市、培育新经济增长点的重中之重来抓。出台了《关于加快民营经济发展的意见》，营造良好的政策环境，降低了营商成本，民营经济走上发展快车道。目前，民营经济已成为我市水泥、石材、不锈钢制品、电子、纺织服装等支柱工业产业和畜牧水产、商贸服务业的主体，是我市经济发展最具活力的亮点。

——城镇化建设有了新发展。2003年，我市城镇化水平达到41%，比1998年提高了10.6个百分点。城镇公共设施不断完善，品位明显提高。尤其是中心城区，通过扩建和完善路网、实施美化绿化亮化工程、增设城市景观、扩大公众活动场所、建设高品位的接待基地和配套设施完备的住宅小区，改善了人居环境，初步搭起了颇具特色的山水城市框架。各县（市）城镇规划编制进一步完善，建设和管理水平不断提高。

——国内外两个市场拓展取得成效。我市在支持企业千方百计增加国内市场份额的基础上，积极扶持企业拓展海外市场。近几年，除香港、澳门、东南亚等市场得到进一步巩固外，还拓展了台湾、日本和欧美市场。在抓好传统贸易的同时，通过大进大出、两头在外等措施，扩大出口额度。市和各县（市、区）在珠三角地区设立了一批招商联络处。兴建了10多个规模较大的工业发展载体，吸纳了一批国内外知名企业到我市投资，增强了发展后劲。

——基础设施建设成绩显著。我市努力加强基础设施建设，增加对交通、能源、水利、通信和城市公共设施的投入，使基础设施建设有了长足的发展。新建和改造了一批县级以上公路以及镇通村公路，完成公路建设改造1679公里，全市通车公路总里程达4229公里，比1998年增加622公里；广梧高速公路马安至河口段将于2004年底建成通车，云城河口至郁南平台段以及至罗定段前期工作基本完成，即将动工建设；云浮发电厂二期和罗定粤泷电厂两台机组相继建成投产，至2003年底，全市发电装机容量达114万千瓦，比1998年增长128%；城乡电网改造工作进展顺利，实现了城乡同网同价目标；建立了政府公众、政务、农村经济、企业信用等信息网站，电子口岸、乡镇财政结算管理、电子公文交换、视频会议等一批应用系统投入使用；电话普及率达到16.1%，比1998年提高了9.9个百分点，实现了“村村通邮”；水利建设进一步加强，抗洪排涝和灌溉能力不断提高，罗定、郁南、新兴成为全国首批农村水电初级电气化县。

——财税金融工作成绩喜人。我市认真落实财政税收各项政策，不断强化税收征管。全面推行收支两条线管理，不断提高科学理财水平，为各项事业发展提供了较稳定的财力支持。金融企业改革取得较好成效，业务得到长足发展，各项存贷款余额不断增加，不良贷款余额大幅减少；积极稳妥地抓好城市信用社和农村合作基金会清理整顿工作，努力兑付了城信社、农金会全部自然人存款和股金，资产保全、追收工作取得了明显成效。

——精神文明建设和民主法制建设得到加强。通过深入开展学习邓小平理论、“三个代表”重要思想和“三讲”教育活动，使政风明显改善；大力开展群众性精神文明创建活动，建设了一批文明行业、文明单位、文明村镇和文明社区。坚持和完善向人大及其常委会报告工作、向政协通报情况的制度，自觉接受监督；加强与工会、共青团和妇联等群众团体的联系，进一步拓宽民主渠道。本届政府出台了规范性文件68件，办理人大议案、建议293件和政协提案471件，受理行政复议案件73件。顺利完成了城市、农村基层体制改革和民主选举村民委员会、社区居委会工作。全面推行村务、厂务和政务公开。强化审计、监察和纠正行业不正之风工作，加大行政执法监督力度，完善行政执法责任制和执法过错责任追究制，行政执法行为进一步规范。大力整顿和规范市场经济秩序，反对行业垄断、反对地区封锁，并查办有关案件。认真做好公证和法律服务工作，通过了省“三五”普法验收。依法加强民族宗教事务管理。重视人民群众来信来访，妥善处理群体性上访事件，保持社会大局稳定。深入开展严打整治斗争，加强社会治安综合治理，开展打黑除恶和扫除“黄赌毒”，严厉打击非法传销、“六合彩”赌博以及“法轮功”邪教等活动，维护了良好的社会治安秩序。

——社会各项事业全面进步。科技创新综合能力和地区竞争力逐步提升。2003年科技进步对经济增长的贡献率由1998年的35%上升到46%。全市科技创新综合能力在全省排第16位。组建了石材和硫化工等5家省级工程中心，创建了省级初城民营科技园、省级农业科技园、省级农业科技创新中心、专业镇技术创新试点和一批省认定的高新技术企业、民营科技企业，有4个产品被评为广东省名牌产品，1个产品获得全国免检产品称号，还有5个商标被认定为省著名商标。

教育事业在改革中快速发展。落实“以县为主”的农村义务教育管理体制，撤销了镇教办，调整和合并了规模小、水平低的教学点，全面开放幼儿教育，教育资源进一步优化，改造山区、老区薄弱学校成效显著。积极推进素质教育，率先在山区市中推行从小学一年级起开设英语课程和全部中学开设信息技术教育课程，“普九”工作不断巩固，普及高中阶段教育速度不断加快，高考上线人数连年增加。积极推进等级示范学校建设，职业技术教育规模不断扩大，创办了罗定职业技术学院，填补了我市高等院校空白。

文化体育工作有了新进步。共投入资金1.8亿元建成文化设施116个，其中市级4个，县级14个，实现了镇镇有文化中心，提前一年完成省人大议案提出的文化设施建设任务，市政府被省评为落实议案的先进单位。罗定市、新兴县被评为国家级文化先进县（市）。坚持主流文化与地域文化、高雅文化与通俗文化、现代文化与传统文化共同发展，文艺创作、文艺演出、书画展等文化活动空前活跃。打造特色文化品牌，举办了两届西江民间艺术节和首届六祖文化节，郁南县连滩镇被命名为中国民间艺术之乡。广播电视事业取得长足发展，广播、电视综合覆盖率分别达到97.4%和95.7%。实施体育“民心工程”，改善城乡体育设施。群众性体育活动蓬勃发展，体育竞技水平不断提高。成功举办了第二届市运会。在省第十一届运动会上，我市代表团总分、金牌数均居全省第15名。

卫生工作取得了新突破。完成了卫生监督和疾病预防控制体制改革，完善了疾病预防控制、医疗救治、卫生监督三大体系。抗击非典工作取得了阶段性重大胜利，2003年我市成为全省6个没有发生非典病例的地级市之一。禽流感疫情得到及时控制。全面开展药品集中招标采购和医疗器械招标采购，降低了医疗成本。农村卫生三项建设和初级卫生保健通过了省的验收。

计划生育、国土资源管理和环境保护工作取得了新的成绩。与1998年相比，全市计划生育率从80.67%上升至89.41%，人口出生率从16.05‰下降至11.07‰，人口自然增长率从10.05‰下降至5.91‰，已进入低生育水平行列。整治了一批石矿场，连续五年实现耕地占补平衡。西江水环境综合治理、南山河整治和困扰市民多年的水泥粉尘、石材废渣、废水的治理工作取得阶段性成效，综合污染指数逐年下降。市城区每平方公里每月降尘量从1998年的10.1吨降至去年的7.8吨，首次达到省的标准。森林资源保护管理继续加强，森林覆盖率达61.4%、活立木总蓄积量为1721万立方米，分别比1998年提高了0.5个百分点和增加261万立方米。安全生产稳中趋好，有效地遏制了特大事故的发生。

——城乡人民生活水平明显提高。2003年在岗职工年平均工资11268元，农民人均年纯收入3798元，5年平均增长分别为10.8%和4.3%。全市城镇人均住房面积20.1平方米，比1998年增加4.7平方米；农村居民人均住房面积21.6平方米，增加6.9平方米。下岗职工基本生活费和企业退休人员基本养老金确保按时足额发放。低保制度逐步健全，保障范围不断扩大。全市五年累计发放低保资金3287万元，共有101万人次享受了低保待遇；共投入救灾资金2711万元，使27.7万受灾人次得到了生活救济。积极解决弱势群体住房、就医、打官司、子女入学等“四难”问题。全面落实就业和再就业政策，城镇登记失业率控制在3.4%以内，广泛开展智力扶贫工程，对城乡贫困人员进行免费技能培训，为脱贫致富打好基础。机关事业单位工作人员福利待遇逐步提高。国防后备力量建设、国防动员、人民武装和双拥优抚工作取得新的成绩。

此外，发展计划、物价、经济发展研究、粮食、统计、人事、档案、人防、地方志、药监、外事、侨务、台务、口岸、消防、保密、新闻出版、妇女儿童、气象等工作也取得了新的成绩。

各位代表，过去五年是我市国民经济持续快速发展、综合实力逐步增强、社会各项事业全面进步、人民生活水平不断提高的五年。取得这样的成绩来之不易。我们的主要体会是：第一，必须高举邓小平理论伟大旗帜，全面贯彻“三个代表”重要思想，紧紧依靠全市广大干部群众，充分调动社会各方面的积极性，上下一心，团结协作，奋发拼搏，形成促进发展的合力；第二，必须坚持从云浮的实际出发，正确处理好长远利益和当前利益的关系，从优化环境、完善制度等基础工作做起，着力解决经济和社会发展过程中的重点、难点问题，夯实发展基础；第三，必须充分认识自己的区位特色，找准定位，选准发展思路，发挥区位、资源优势，在参与地区分工尤其是接受珠三角地区产业转移中寻求发展；第四，必须坚持解放思想，与时俱进，敢为人先，大力推进体制创新，增强发展动力；第五，必须坚持科学发展观，以人为本，促进经济和社会各项事业全面、协调发展，走可持续发展之路。

各位代表，我市过去五年取得的成绩，是在党中央、国务院，省委、省政府和市委的正确领导下，全市人民迎难而上、积极进取的结果，是各级人民代表大会及其常务委员会和人民政协监督支持的结果。在此，我代表市人民政府，向全市广大工人、农民、知识分子、干部，驻云浮解放军、武警部队、消防官兵和公安干警以及各界人士表示崇高的敬意！向关心和支持云浮建设的香港、澳门特别行政区同胞、台湾同胞和海外侨胞以及一切国际友人表示衷心的感谢！

回顾过去的五年，我们也清醒地看到，我市经济和社会发展中仍然存在不少困难和问题。主要是：经济总量较小，结构不合理；二、三产业发展滞后，规模企业少，经济外向度低；中心城区的经济实力和辐射带动能力有待进一步增强；农民增收缓慢，就业和再就业压力较大，城乡低收入群体生活困难；社会上存在一些影响稳定的因素，维护稳定的任务还很重；机关作风有待进一步改进，公务员素质有待进一步提高。对这些困难和问题，我们必须高度重视，进一步采取有力措施，逐步加以克服和解决。

今后五年的奋斗目标和发展思路

各位代表，今后五年是我市经济和社会发展的重要时期。中共中央政治局委员、省委书记张德江去年视察云浮时，明确提出要把云浮建设成为山区经济强市，到2020年，与全省同步全面建成小康社会，率先基本实现社会主义现代化，这是省委、省政府对我们寄予的殷切期望，也是全市人民的共同愿望。为了实现这一宏伟目标，我们要振奋精神，努力工作，在市委的领导下，团结和带领全市人民，艰苦奋斗，开创我市经济建设和社会发展的新局面。

根据市第三次党代会精神，今后五年政府工作指导思想是：高举邓小平理论伟大旗帜，以“三个代表”重要思想为指导，认真贯彻党的十六大、省第九次党代会以及张德江同志视察云浮重要讲话和市第三次党代会精神，以建设山区经济强市为总目标，坚持以人为本，树立和落实科学发展观；坚持求真务实，走珠三角经济区延伸发展路子；在继续实施“一城崛起、三线带动、五星争辉”的基础上，构筑“东渐西联，优内拓外，错位竞争，统筹并进”的发展新格局，加快完善社会主义市场经济体系，全面提升经济实力和综合竞争力，努力改善人民生活，加强民主法制建设，依法行政，推进廉洁高效政府建设，促进三个文明协调发展，跟上全省率先基本实现现代化的步伐。

经过五年奋斗，基本形成与山区经济强市相适应的经济规模和综合实力。到2008年，全市地方生产总值和地方财政一般预算收入翻一番。

为了实现上述目标，我们要抢抓机遇，积极贯彻落实市第三次党代会精神，努力做好各方面工作。

（一）实施东渐西联，走珠三角经济区延伸发展路子，积极参与泛珠三角区域合作。充分发挥我市地处泛珠三角延伸主轴和连接带的有利区位优势，加快构建联接珠三角平台，加大吸纳产业转移力度，建设好珠三角的产业转移基地、生产配套基地、农副产品供应基地和劳动力输出基地，加快调整经济发展规划和产业布局，使我市经济发展与珠三角经济区更加紧密地联成一体; 加强与泛珠三角西部区域的联系与合作，参与经济大循环，进一步加快我市的经济发展。

（二）实施优内拓外，营造良好的发展环境，拓展国内外两个市场。加强基础设施建设，按照适度超前的原则，高质量、高标准建设一批交通、能源、通信、水利等基础设施项目。重点抓好高速公路、西江黄金水道的规划和建设，力争2008年实现县县通高速公路。抓好中心城区、各县（市）城区和中心镇的规划建设。推进廉洁高效政府建设，增强依法行政意识，进一步改进机关作风，提高工作效率和办事质量，切实改善吸引外来投资的软硬环境，进一步拓展国内外市场，积极拓宽利用外资渠道，全方位招商引资。

（三）实施错位竞争，走特色发展之路，壮大区域经济实力。立足地方特色，发挥比较优势，优化生产力布局。适应市场经济需要，根据专业化和市场分工，提供自己的特色产品和配套服务。通过五年的奋斗，把中心城区建设成为具有较强综合服务功能和辐射带动能力的区域性经济中心，各县(市)成为具有明显地方特色的山区经济强县。加快新型工业化、城镇化和农业产业化建设，突出抓好西江沿江经济带和高速公路经济带的规划和建设，将其发展成为集现代物流和工业于一体的新型经济发展载体；着力打造水泥生产基地、石材生产基地、不锈钢制品生产出口基地、农产品种养加工基地和电力电子工业基地。

（四）实施统筹并进，落实科学发展观，推动社会各项事业全面进步。不断推进社会主义物质文明、政治文明和精神文明建设。加强公民道德建设，提高公民的思想道德水平和科学文化素质。加强国防教育，增强国防观念。大力发展科技、教育、文化、卫生、体育等各项社会事业。严格执行计划生育、保护环境和保护资源的基本国策，走经济持续发展、社会全面进步、资源永续利用、环境不断改善、生态良性循环的新型发展道路。关心群众生产生活，落实就业和再就业政策，加大扶贫开发和农村富余劳动力转移力度，增加城乡居民收入。建立健全与经济发展水平相适应的社会保障体系，让全市人民共享经济和社会发展成果。

各位代表，我市今后五年的奋斗目标和发展思路已经明确。新一届政府肩负着建设山区经济强市的使命，承载着全市人民的重托，我们要以十六大精神为指导，全面贯彻“三个代表”重要思想，努力建设学习型、创新型、法制型、服务型、廉洁高效型政府。勇于探索，不断提高科学决策、统揽全局和驾驭市场经济的能力。进一步加强公务员队伍建设，提高理论素养和工作水平；把发展作为第一要务，聚精会神抓好经济建设和社会事业发展。以宪执政，依法行政，在继续抓好经济调节、加强市场监管的同时，强化政府履行社会管理公共服务职能；改进作风，执政为民，树立科学发展观和正确的政绩观，求真务实，将更多的精力深入基层，关注民生，为群众多办好事实事，把人民群众的利益维护好、实现好、发展好。增强工作透明度，提高政府公信力；深入开展廉政建设和反腐败斗争，树立勤政、廉政、优政的良好形象，扎实做好新一届政府的各项工作。

2004年工作安排

各位代表，2004年是新一届政府的开局之年，做好今年工作，对于实现今后五年奋斗目标具有十分重要的意义。2004年，全市经济和社会发展的预期目标是：地方生产总值增长13%，社会消费品零售总额增长13%，固定资产投资增长40%，地方财政一般预算收入增长13%，外贸出口总值增长10%，实际利用外资增长16%，居民消费价格总指数上升0.5个百分点，城镇登记失业率控制在3.3%以下，人口自然增长率控制在7.7‰以内，农村居民人均纯收入增长3.7%。

围绕上述目标，必须做好以下工作：

（一）以增加农民收入为重点，促进农村经济发展。

加强农村集体土地管理，完善土地征用制度，切实保护基本农田；落实种粮大户生产补贴，千方百计稳定粮食生产。抓好农业现代化示范区和优势农产品示范基地建设，引导农民进一步优化种养结构，大力发展无公害农产品、绿色食品、有机食品和其他优质高值产品。大力推进农业产业化，积极扶持农业龙头企业和农民专业协会发展，继续推广“公司+农户”的温氏模式，增强辐射和带动作用。扶持以批发市场为重点的农产品市场体系和流通中介组织的发展，促进农产品加工流通业，提高农产品的附加值。加强农民职业技能培训，提高科技水平和就业能力，促进农村富余劳动力转移就业，发展劳务经济。抓好中心镇规划修编和建设工作，继续加大扶贫开发力度，切实推进“千村扶贫工程”，促进农村经济全面协调发展。

（二）狠抓项目建设，加快工业发展步伐。

去年以来，我市各地掀起了建市以来少有的投资热潮，项目建设方兴未艾，今年要继续加温鼓劲。已建成的项目，要抓好达标达产，尽早出效益；对在建的项目，要加快工程进度，争取早竣工投产；已签约未动工的项目，要组织力量跟踪落实，做到签约一个成功一个；对一些投资大的项目，要由领导牵头，组成专门工作小组，帮助投资者抓紧建设。同时，要通过启动沿江经济带和高速公路经济带及其他引资载体，增加一批发展前景好，对当地经济有较大影响的项目。此外，还要引导和鼓励现有企业增资扩产或运用新技术和新工艺进行技术改造，扩大生产规模，提高产品档次，增加经济效益。

要认真贯彻我市《关于加快民营经济发展的意见》，全面落实加快民营经济发展的政策措施，创造良好的政策、政务、市场、法制和舆论环境，推进民营企业制度创新、技术创新、管理创新，培育具有较强竞争力的民营企业。要积极帮助民营企业解决资金和技术困难等问题。与此同时，各地要狠下决心，在今年内全部完成国有企业转制任务。通过改革、盘活资产，催生一批充满活力的民营企业。

（三）提高对外开放水平，发展壮大外源型经济。

开拓国际市场。优化外贸出口商品结构，力争培育一批出口名牌和著名商标。提高出口产品的科技含量和附加值。巩固现有市场，大力发展多元化国际市场。扩大一般贸易出口，提高加工贸易水平，发展服务贸易。支持民营企业扩大对外经贸和对外承包工程以及劳务合作业务。

扩大直接利用外资规模。继续坚持招商引资重心下移方针，积极运用多种形式扩大招商引资，大力发展“三资”和“三来一补”企业。继续扶持和发展重点地区、重点企业、重点产品、重点市场的出口。积极应对出口退税政策调整，为符合条件的企业申报办理进出口经营权，进一步深化外经贸体制改革。

（四）以高速公路建设为重点，进一步加快基础设施建设。

今年，要确保广梧高速公路马安至河口段年底通车，云城至郁南平台段及罗定段全线动工，力争做好思劳至新兴支线前期工作；启动西江黄金水道规划和建设，抓好省道352线罗定段、113线新兴段改造以及新世纪大道建设，全面完成在建的27个通镇公路改造项目，加快镇通村公路建设。加强水资源管理及三防工作，实施沿江堤围加固达标和城乡水利防灾减灾工程，落实省人大关于小型水库除险加固、解决水库移民遗留问题、机电排灌及小水电等议案。抓好云浮发电厂三期、粤泷电厂二期以及华润电力等重大项目的立项工作和第三期农村电网改造。

抓好中心城区规划建设。加快完善路网架构，改造美化出入口，扩大城区规模。抓好云浮中学新校区、市党校、城区生活污水处理厂、生活垃圾处理场和消防设施等公共项目建设。继续推进城区美化、绿化、净化、亮化工程，进一步提高城市品位。鼓励和引导民资进入能源、交通、环境保护等基础性设施及公用事业，参与一般竞争性项目建设。

（五）加快发展第三产业，培植新的经济增长点。

要利用六祖惠能、邓发、蔡廷锴等人文资源和国恩寺、蟠龙洞等历史、自然资源，丰富旅游的文化内涵，加快我市旅游产业的发展。因地制宜，规划和建设商业网点，扶持发展连锁经营企业、物流企业，发展各类专业市场；大力发展房地产业、中介业和服务业，刺激住房、汽车、通信、教育和卫生保健等消费热点，推动消费升级，增加消费总量。

（六）加快发展科教事业，为建设山区经济强市提供人才和智力支持。

完善科技创新体系，增强科技创新能力。深化科技体制改革，巩固和完善以企业为主体的创新体系，引导企业建立技术开发机构。加强科技攻关，推进高新技术的开发和应用，创办民营科技园和农业科技示范基地。重视专业技术人才队伍建设和人才资源整体开发。扎实开展科普教育，加强科技服务，推进科技成果转化。依法保护知识产权。

推进教育创新，加快教育改革发展。巩固提高“普九”工作，抓好中小学校布局调整、薄弱学校改造和规范化学校建设。加快建设普通高中学校，增加优质学位，力争2007年基本普及高中阶段教育。进一步加强教师队伍建设。推进中小学信息技术教育和英语教育，实现小学一年级以上开设英语课，所有中心小学、县城小学以及规模较大的完全小学从四年级起开设信息技术教育课程。大力发展职业技术与成人教育，培养高素质劳动者和实用型人才，市技工学校要在今年动工建设，明年9月份投入使用。

（七）扩大就业和提高社会保障水平，改善人民生活。

全面落实就业和再就业扶持政策，努力增加就业岗位，实施公益性岗位申报制度，同时加大就业培训力度，促进我市就业和再就业工作发展。加强劳动监察和劳动关系协调，保护劳动者合法权益。社会保险在今年7月实行市级统筹后，各级政府要继续对社会保险扩面和保费征收工作负责。要强化社会保险基金的监管和待遇核发工作，确保基金安全。企业退休人员基本养老金按时足额发放，推进企业退休人员社会化管理服务工作。

继续完善社会救助制度，实施好“十项民心工程”，切实帮助困难群众解决在医疗、法律援助和子女上学等方面遇到的困难，力争在2005年基本解决城镇特困居民住房困难问题，并尽早完成农村危房改造。关心和支持残疾人就业。进一步完善优抚、“五保”和城乡最低生活保障制度，对符合条件的城乡困难居民实行应保尽保。启动城市流浪乞讨人员救助机制，使他们的基本生活得到保障。

（八）抓好财税金融工作。

加快税收信息化步伐，构建协税护税网络。强化税收征管，扩大私营企业、个体工商户建帐覆盖面。严厉打击各种偷逃骗抗税行为，清缴欠税，做到应收尽收，但不收过头税。大力培植税源，提高财政收入占地方生产总值的比重。调整优化支出结构，严格预算管理，增加教育、科技、环保、医疗卫生和社会保障等社会公共服务的投入，加大对农村社会事业的财政支持。健全公共财政体制。做好实施国库集中收付制度的准备工作，推进“金财工程”建设，巩固政府招标采购和收支两条线改革成果，开展绩效预算改革试点，全面推行部门预算，提高防范财政风险能力。

加强金融服务，增强金融对经济发展的支持力度，规范发展消费信贷。依法处置地方金融不良资产和政府债务，稳妥推进农村信用社体制改革，做好地方金融风险防范和化解工作。鼓励扩大证券、保险等业务。建立企业信用评价、监督管理、责任追究等信用制度，促进企业守法经营。建立规范的个人信用制度，加强公民的诚信意识。

（九）统筹经济社会协调发展，推进社会各项事业。

加强环境治理，提高环境质量。进一步落实各级政府环保目标责任制。加强西江流域整治，重点保护饮用水源，解决好缺水地区人畜饮水困难问题。继续实施治污保洁工程，抓好重点污染源治理。加强建设项目环保管理，从源头上控制新污染源的产生。

加强生态资源保护。在全市推广云城安塘斗带生态试点村经验，抓好100个生态村建设。加强林业生态建设和资源管理，大力发展非公有制林业。倡导、扶持使用可再生能源，依法保护和合理开发自然资源，健全国有资源有偿使用制度。

提高人口与计划生育工作水平。落实人口与计划生育目标管理责任制，推进计划生育村民居民自治，实行流动人口以现居住地管理为主的工作机制。推进农村计划生育家庭奖励扶助制度。积极开展关爱女孩工作，综合治理人口性别比升高问题。稳定低生育水平，提高人口素质。做好老龄人口工作。

加强文化建设，抓好市图书馆、博物馆和档案馆等文化基础设施建设，多出文化精品，拓展文化产业，繁荣文化市场。全面完善市、县、镇广播电视光纤网络，完成大绀山广播电视发射塔建设，实现广播电视多重优质传输覆盖。加强疾病预防控制、卫生监督执法和医疗救治三大体系建设，提高公共卫生保障水平。深化城镇医疗卫生体制改革，做好重大疾病防治。加强农村卫生工作，巩固和发展新型农村合作医疗和农民住院补充医疗保险。完善药品集中招标采购制度。继续实施体育民心工程，积极开展全民健身活动，抓好参加省第十二届运动会准备工作。努力增加城乡体育设施，搞好市体育公园规划。尊重和保护公民宗教信仰自由，依法加强宗教事务和少数民族工作。进一步做好海外侨胞、港澳台同胞和侨属工作。实施妇女儿童发展纲要，维护妇女儿童合法权益，加强未成年人思想道德建设，优化成长环境。抓好全民国防教育，做好民兵预备役工作，加强双拥共建和优抚安置工作，继续重视和做好外事、人防、统计、档案、方志、口岸、气象等工作。

推进民主法制建设和精神文明建设，建立社会主义政治文明。坚持政府向人大及其常委会报告工作，向人民政协通报情况的制度，自觉接受监督。认真办好人大议案、建议和政协提案。多方听取社情民意。推进政务、厂务、村务公开。加强农村基层政权建设，完善村民居民自治及职工代表大会等民主管理制度。贯彻国务院全面推进依法行政实施纲要，加强政府法制建设，强化行政执法监督，完善行政复议制度，提高依法行政水平。继续加强普法工作，实行依法治市和以德治市相结合。加强对广大干部群众的思想道德、民族精神、爱国主义和艰苦奋斗教育，扎实开展各类精神文明创建活动。深入实践“三个代表”重要思想，为经济建设和社会发展提供强大精神动力。

规范市场经济秩序。积极发展产权、土地、劳动力和技术等要素市场，规范市场主体资格和市场行为。反对地区封锁、行业垄断和价格垄断等不正当竞争。完善适应社会主义市场经济的信用制度。强化专项整治，严厉打击制售假冒伪劣商品、传销和变相传销、做假帐、商业欺诈、侵犯知识产权、非法洗钱等各种破坏市场经济秩序的违法犯罪行为。实施食品药品放心工程。

加强安全生产管理。全面贯彻安全生产法，进一步落实安全生产责任制和重特大安全事故行政责任追究制度，建立完善应急救援体系。加强宣传教育，大力开展专项整治，排除事故隐患，预防重特大安全事故的发生。

抓好社会稳定各项工作。落实社会治安综合治理目标管理责任制、领导责任制和一票否决等制度。扫除黄、赌、毒、黑等社会丑恶现象，坚决打击“法轮功”邪教组织和“六合彩”赌博活动，严厉打击各种刑事犯罪和经济犯罪。重视信访工作，落实信访包案责任制，妥善处置群体性事件，把纠纷化解于萌芽状态。增强公民国家安全意识，做好国家安全工作。

各位代表！过去五年，我们取得了辉煌成就；展望未来，我们豪情满怀，充满信心。让我们高举邓小平理论和“三个代表”重要思想伟大旗帜，以党的十六大精神为指导，紧密团结在以胡锦涛同志为总书记的党中央周围，在市委的领导下，解放思想，与时俱进，开拓进取，同心同德，为建设山区经济强市，全面建设小康社会而努力奋斗！
